# Acute Physical Exercise Reduces Mental Stress-Induced Responses in Teachers

**DOI:** 10.3390/ijerph22060924

**Published:** 2025-06-11

**Authors:** Laura Cristina Nonato, Alexandre Konig Garcia Prado, Daniela Lopes dos Santos, Karen Dennise Lozada Tobar, Jaqueline Alves Araújo, Jacielle Carolina Ferreira, Lucieli Teresa Cambri

**Affiliations:** 1 Postgraduate Program in Physical Education, Federal University of Mato Grosso, Cuiabá 78060-900, Brazil; lauranato@hotmail.com (L.C.N.); akgprado@gmail.com (A.K.G.P.); dennise_lozada@hotmail.com (K.D.L.T.); jackellynne_alves@hotmail.com (J.A.A.); jacielleferreira@gmail.com (J.C.F.); 2 Physical Education and Sports Center, Federal University of Santa Maria, Santa Maria 97105-900, Brazil; lopesdossantosdaniela@gmail.com

**Keywords:** aerobic exercise, blood pressure, cardiovascular reactivity, heart rate variability, obesity, reproducibility, Stroop color word test

## Abstract

This study assessed the correlation between obesity markers and mental stress reactivity. Mainly, it evaluated whether physical exercise (PE) influences cardiovascular reactivity to the Stroop color word test (SCWT) in teachers. Thirty-one school teachers were evaluated. The SCWT was carried out under (1) baseline and (2) 30 min after aerobic PE conditions. Teachers performed 30 min of PE. The reactivity to mental stress (Δ) during the SCWT for blood pressure (BP) was determined, with Δ being the highest value observed [Δ2 or Δ4: with pre-test values (0 min)]. Of the teachers, 64.52% were considered overweight/obese and 19.35% had a high clinical BP. However, 67.74% of teachers were considered physically active. Systolic BP (SBP) reactivity to SCWT correlated negatively with obesity markers (Rho = −0.36 to −0.60; *p* < 0.05). The SBP and diastolic BP (DBP) were higher at 2 and 4 min compared to 0 during the SCWT under both conditions (*p* < 0.01). Moreover, SBP was always lower after PE (*p* < 0.01) and DBP was lower at 2 and 4 min after PE (*p* < 0.01). In summary, SBP reactivity to mental stress correlated negatively with obesity markers. Moderate-intensity acute PE reduced BP reactivity to mental stress in teachers.

## 1. Introduction

The interplay between obesity and cardiovascular disease outcomes in specific subpopulations (i.e., teachers) is complex and requires further elucidation. An additional risk factor for cardiovascular disease is occupational stress, common in professions such as teaching [1]. Studies have reported the association between exaggerated cardiovascular reactions (i.e., blood pressure—BP and heart rate—HR) and acute psychological stress and risk for future hypertension [2,3]. Stress evaluation in the laboratory is commonly conducted using the Stroop color word test (SCWT) [4].

In this context, one strategy for BP control is to perform physical exercise, since even a single exercise session can help in BP control [5]. Thirty minutes of physical exercise (PE) may have both a protective cardiovascular effect during the daily teaching routine and a more significant BP decrease during sleeping [6]. Exercise before work might be an interesting strategy for cardiovascular protection in academic professionals.

A recent meta-analysis showed that acute PE lowers BP reactivity in response to stressor tasks. These results, however, referred mainly to healthy younger adults (a mean age of less than 30 years), who are over-represented in many studies [4], and few studies used the SCWT as a stressor [7]. Likewise, studies involving teachers have evaluated obesity indicators, BP, and the relationship between unhealthy habits such as physical inactivity [1,8,9,10,11,12]. Few of these studies [6], however, have considered the influence of PE as an acute and chronic prevention against negative cardiovascular outcomes in this population. As far as we know, no research has considered BP reactivity to mental stress in specific populations, such as teachers, much less these responses to acute aerobic exercise. This evidence would allow more satisfactory health management geared towards avoiding the development of hypertension in the teaching profession, with high ecological validity. The findings of the present research may be fundamental for defining adequate clinical approaches for the prevention and non-pharmacological treatment of hypertension, aiding teachers to maintain good physical and mental health to meet the care requirements of their students.

The purpose of this research was to evaluate the correlation between obesity markers and cardiovascular reactivity to mental stress. Mainly, it was to analyze whether PE influences cardiovascular reactivity to mental stress in teachers. We hypothesized that acute aerobic PE promotes lower cardiovascular reactivity to mental stress in teachers.

## 2. Materials and Methods

Procedures were carried out in the city with an estimated population of 16,999 inhabitants and a Human Development Index of 0.65. There are three state schools in the city with a total of 92 teachers. We visited the schools and invited the teachers to participate in the research. All 92 teachers were interviewed, and 40 met the inclusion criteria; however, three did not agree to participate in this study and six withdrew from the study due to lack of time. The study included 31 teachers. Data collection was carried out in the school environment at days and times scheduled in advance. The inclusion criteria were: adults of both sexes, aged 18 to 50 years, body mass index (BMI) range 18.5 ≤ 39.9 kg∙m^−2^, active school teachers, with at least two years in the classroom, and having no regular PE. The exclusion criteria were: color blindness, smokers, use of any substances that could interfere with the variables under study (BP and HR variability—HRV), presenting osteoarticular problems that would make PE impossible. Pregnant or breastfeeding women were excluded, as well as teachers who were on leave, on vacation or working as Director or Pedagogical Coordinator. The protocol study was conducted in accordance with the standards set by the Declaration of Helsinki and the ethical guidelines of the Ethics Committee in Human Research of the Federal University of Mato Grosso. All subjects provided written informed consent to participate this study.

### 2.1. Study Design

**Figure 1:**
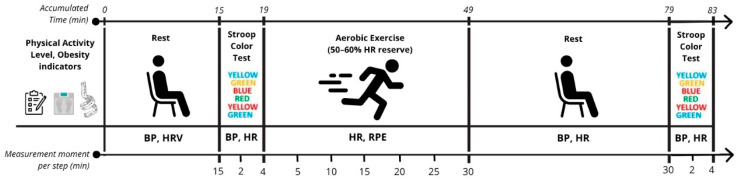
Study design. BP: blood pressure; HR: heart rate; HRV: heart rate variability; RPE: rate of perceived effort.

After filling in a health history and substance-use questionnaire to validate the inclusion criteria, determination of physical activity level, and anthropometric measures, teachers took part in an experimental session. They started with baseline measurements in a seated position for 15 min. Then, the teachers were subjected to the SCWT both before (baseline) and 30 min after aerobic PE—Figure 1. The dependent variables of our study were BP and heart rate. The independent variables were SCWT and a physical exercise session.

### 2.2. Experimental Session

The teachers were instructed not to engage in vigorous-intensity activities and not to drink alcoholic beverages or stimulants (i.e., coffee, tea, and soft drinks) or any other substances that could potentially alter the autonomic nervous system for 24 h before the evaluations. Furthermore, the teachers were instructed not to eat 1 h before the beginning of the evaluations. The evaluations were conducted between 6:00 a.m. and 12:00 p.m., always by the same evaluator.

### 2.3. Level of Physical Activity

The teachers were instructed to answer a questionnaire based on activities undertaken during the previous week. Level of physical activity was measured through the International Physical Activity Questionnaire (IPAQ), short-form. Teachers were considered as physically active when they reached the recommendations, with a minimum 150 min·wk^−1^ of moderate-intensity or at least 75 min·wk^−1^ of vigorous intensity. The other teachers were considered as insufficiently active [13].

### 2.4. Obesity Markers

To determine BMI, body mass (OMRON Corporation, Kyoto, Japan) and height (Sanny stadiometer, 0.1 cm, Rio de Janeiro, Brazil) were evaluated. Moreover, bioimpedance (OMRON Corporation HBF-514C, Kyoto, Japan) was used to estimate the percentage (%) of body fat. We followed the manufacturer’s instructions, instructing the teachers to empty their bladders and not ingest any liquids 30 min before the bioimpedance measurement. In addition, abdominal circumference was measured (Cardiomed tape; 0.1 cm, Rio de Janeiro, Brazil). The teachers were classified according to BMI: normal weight (18–24.9 kg·m^−2^), overweight (25–29.9 kg·m^−2^) and obese (≥30 kg·m^−2^) [14]. High abdominal circumference was considered as ≥80 cm for women and ≥94 cm to for men [15].

### 2.5. Blood Pressure and Heart Rate Variability

BP was measured three times with an automatic sphygmomanometer (Microlife, BP 3BT0-A^®^, Rio de Janeiro, Brazil), with a one-minute interval between measurements and after resting for 15 min. The individual was sitting, with feet flat on the floor, back supported, and arm with cuff supported at heart level. The value considered was based on the average of the two last measures. The teachers were classified as having high BP with Systolic BP (SBP) ≥ 130 or diastolic BP (DBP) ≥ 85 mmHg values [16]. For the determination of the HRV, the RR intervals (RRi) were collected continuously during the final 5 min of rest, with an HR monitor (Polar model V800, Kempele, Finland). Data acquired with this monitor have been shown to have a good reproducibility [17]. Breathing rate was not controlled, as doing so would perturb the natural breathing pattern.

### 2.6. Stroop Color Word Test—SCWT

The SCWT was performed at the end of the baseline and 30 min after completion of 30 min of moderate aerobic exercise. A panel with the names of four colors (blue, red, green, and yellow) each had the name of a color written with letters of a different color. This was shown to the teachers over four minutes and the words were altered each second. To increase the mental stress level, an auditory conflict with different names of colors was added. The teachers had to say the color of the letters without reading the word, as quickly as possible [18]. Each teacher was asked to evaluate their difficulty in performing the test conforming to previously established levels of difficulty. We used two tests (test 1, and test 2) with different color sequences at baseline and post PE, to avoid the learning effect. We achieved balance by using a random order for the tests. BP and HR were recorded at minutes two and four during the SCWT.

A pilot study was carried out prior to the collection of main data to evaluate the reproducibility of the SCWT. Twelve other participants (5 women and 7 men; 25.69 ± 4.83 years old; 72.43 ± 16.88 kg; 24.24 ± 4.02 kg·m^−2^) performed the SCWT twice with a 30 min interval between then. There was no significant difference (*p* < 0.05) between the test and retest for BP and HR. All variables presented high to very high intraclass correlation coefficients (ICC), and a low coefficient of variation (CV) and standard error of measurement (SEM)—Table 1. The results show that the test seems to be reproducible.

### 2.7. Physical Exercise

Teachers were requested to walk for 30 min at 50–60% of reserve HR (HR max−HR rest) in an external environment. Rate of perceived effort (RPE) was performed using the 20-point Borg scale [19]. RPE and HR were monitored every 5 min during the 30 min of PE. After PE, the teachers stayed in a seated position for 30 min, and BP and HR were measured at the end of this time.

### 2.8. Data Analysis

HRV indices (Kubios Oy, Version 2.1, Kuopio, Finland) were evaluated using the RRi (Polar model V800, Kempele, Finland) during the last 5 min interval at baseline condition. We filtered the artifacts using the software’s moderate filter. The HRV indices were determined using spectral analysis and an autoregressive algorithm model at order 16. The HRV indices evaluated were: (1) nonlinear: determined through Poincaré plots, standard deviation (SD) of the instantaneous RRi (SD1), and SD of the long-term RRi (SD2); (2) linear time-domain indices: root mean square of adjacent RRi differences (RMSSD); the SD of normal-to-normal RRi (SDNN) and the relative number of successive RRi that differed in duration more than 50 ms (pNN50). (3) Frequency-domain indices: low frequency (LF, with a 0.04–0.15 Hz variation); high frequency (HF, with a 0.15–0.4 Hz variation, expressed in n.u.) and the LF/HF ratio. The power of each spectral component was normalized by dividing the power of each spectrum band by the total power minus the value of the very-low-frequency band value and multiplying by 100. The SD1, RMSSD, pNN50 and HF n.u. indices represent the parasympathetic modulation. The HF index, also called the respiratory band, corresponds to the HR variations related to the respiratory cycle. The SD2 and SDNN indexes represent the global variability. The LF n.u. index is mainly related to baroreflex control by both parasympathetic and sympathetic modulation.

The reactivity to mental stress (Δ) at 2 and 4 min during the SCWT under baseline and after-PE conditions, for BP and HR, were determined as being: Δ: 2 and 4 min, with pre-test values (0 min) considering the highest Δ value observed (Δ2 or Δ4). The BP and HR values at 30 min after PE were taken as the pre-SCWT values.

### 2.9. Statistical Analysis

The Shapiro–Wilk test was applied to check the data normality. Parametric data were shown as mean and standard deviation, and nonparametric data were shown in median (minimum—maximum values). The reactivity to mental stress (Δ) between the conditions (baseline vs. after PE) was compared through the paired Student’s *t*-test of parametric data or the Wilcoxon test of non-parametric data. The obesity markers, BP, HRV indices and cardiovascular reactivity to SWCT at baseline and after PE were correlated through Pearson’s linear correlation (r) to parametric data, and Spearman’s Rank (Rho) to non-parametric data; an r higher than 0.1, 0.3 and 0.5 was considered to have a small, medium or large effect. To compare cardiovascular responses to mental stress at baseline and after PE, a two-way repeated measures ANOVA (2 × 3) was utilized, considering condition (baseline/after PE) and time during SCWT (0, 2 and 4 min). We utilized Bonferroni’s post when there was an interaction between factors. Effect size (ES) measures of the ANOVA factor analysis were determined by the partial eta square (ƞ^2^). The statistical power (Pr = 1 − β) to a posteriori of the main analysis was presented. The significance level considered was *p* ≤ 0.05.

## 3. Results

The teachers showed high obesity markers, with 64.52% of them considered overweight/obese, and only 35.48% of normal weight. In addition, 58.06% of teachers were considered to have a large abdominal circumference; 6.21% reported a hypertension diagnosis and 19.35% had high clinical BP. However, 67.74% of teachers were considered physically active, with only 32.26% considered insufficiently active (Table 2).

During the 30 min of PE, the teachers walked ~3 km at 6 km·h^−1^ with an HR of 132 ± 8 bpm and RPE of 10 (Table 3). A single exercise session reduced SBP (111 ± 10 vs. 114 ± 10 mmHg; *p*: 0.02) 30 min after PE, but did not reduce DBP compared to baseline.

The SBP (12 ± 12 vs. 17 ± 10 mmHg, *p* = 0.01) and DBP (7 ± 9 vs. 12 ± 6 mmHg, *p* < 0.01) reactivity to mental stress reduced after PE compared to baseline. However, HR reactivity (9 ± 8 vs. 10 ± 6 bpm, *p* = 0.43) to mental stress did not change after PE. SBP reactivity to stress correlated negatively (*p* < 0.05) with body mass and BMI both under baseline conditions (Rho = −0.38 and −0.38) and after acute PE (Rho = −0.48 and −0.36). HR reactivity to stress correlated with body mass (Rho = − 0.39; *p* < 0.05), both under baseline conditions, and with body mass, BMI, and abdominal circumference (Rho = −0.63; −0.60; −0.39) after acute PE. Abdominal circumference and SBP (Rho = −0.39 and −0.38) correlated negatively (*p* < 0.05) with the pNN50 index. However, there were no significant correlations between BP reactivity to stress with the HRV indices.

When the mental stress test was performed under the post-PE condition, it caused similar hemodynamic responses as baseline, including increased SBP, DBP, and heart rate. However, SBP and DBP had lower absolute values despite the higher heart rate. For SBP, there was a main effect of time and condition, as well as a significant interaction between time and condition (time x condition interaction, *p* < 0.01; Pr = 0.76). The SBP was higher at 2 and 4 min compared to 0 during mental stress under both conditions (*p* < 0.01; Pr = 1.00). Furthermore, SBP was lower for the after-PE condition at all times (*p* < 0.01; Pr = 1.00)—Table 4.

For DBP, there was a main effect of time, as well as a significant interaction, between time and condition (time × condition interaction, *p* = 0.01; Pr = 0.92). The DBP was higher at 2 and 4 min compared to 0 during mental stress under both conditions (*p* < 0.01; Pr = 1.00). Moreover, DBP was lower under the post-exercise condition at 2 and 4 min after PE.

Heart rate was higher (time main effect; *p* < 0.01; Pr = 1.00) at 2 and 4 min compared to 0 during mental stress, regardless of condition. Additionally, HR was higher (condition main effect; *p* < 0.01; Pr = 1.00) post-exercise compared to the baseline condition, regardless of time (Table 4).

There was no difference in the difficulty of the task perceived during the SCWT between conditions [baseline: 2 (0–4) vs. after PE: 1 (0–4).]

## 4. Discussion

The main conclusions of this research were that (a) SBP and HR reactivity to mental stress correlated negatively with obesity markers; (b) moderate-intensity aerobic PE reduced BP reactivity to mental stress. The highlight of this research was to explore the acute exercise cardioprotective role in stress reactivity in populations that are likely to present high occupational stress.

Our results demonstrated that school teachers presented significantly higher rates of overweight (64.52%) compared to the general population in the State Capital (Cuiabá, 57%) or the Brazilian (57.2%) reference population [20]. However, these rates are similar to other studies involving teachers [21,22]. About 20% of the teachers in our study had high BP, lower than in some studies in different countries (i.e., 29 to 35%) [9,23]. Teacher activities can influence high BP values that may be a result of the high psychological stress levels and sitting time experienced in this occupation [9,10]. In the present research, abdominal circumference and SBP were significantly correlated with the pNN50 index, agreeing with previous findings in the general population [24,25] and subgroups such as policemen [26].

Although having no regular PE had been an inclusion criterion, 67.74% of the teachers evaluated were considered active, a higher rate than the general Brazilian population [20]. We speculate that the high level of physical activity observed may be since the study was conducted in a small town, where most teachers live close to the schools and often commute on foot or by bicycle. The physical activity level seems to have been insufficient to keep the obesity markers at acceptable levels. However, teaching involves sedentary behavior most of the time, and long work shifts which have already been addressed as an independent health risk [10]. This parameter, however, was not part of the present research. Some of the ways that the work facilitates weight gain such as psychological stress, long working hours and overtime, could result in fatigue and reduce behaviors that increase energy expenditure. These could lead to a modification of endocrine factors, and behaviors such as sedentary leisure activities [27].

In our research, the BP and HR reactivity to the SCWT correlated negatively with obesity markers under both the baseline and after-PE conditions. In a recent study, the authors observed similar data [18]: obese individuals presented lower BP reactivity to SCWT; and their reactivity to SCWT correlated negatively with obesity markers at baseline conditions. These findings provide further support for an inverted-U model of the reactivity hypothesis, in which intermediate responses may be the healthiest responses, but exaggerated and blunted responses may be detrimental [28]. Exaggerated cardiovascular stress reactivity has been related to high BP and hypertension risk [2,3,29]; and blunted stress reactivity has been related to obesity markers and obesity incidence [28,30]. Obesity measures have been significantly negatively associated with HR and cortisol reactivity [30]; and in a prospective study over a 5-year period, low HR reactivity was associated with increased risk of becoming obese [31].

The reduction in BP post-exercise has important clinical significance [32]. Furthermore, BP stress reactivity was attenuated by acute PE. Similar to a previous study, an aerobic PE session moderated SBP reactivity to mental stress, regardless of obesity [18], and also in women with rheumatoid arthritis and hypertension [33]. Another study found the same: the BP response to mental stress was attenuated after a single session of maximum exercise in normotensive individuals, an effect that occurred with lower stroke volume and cardiac output [34]. Since both reduced SBP and DBP reactivity post-exercise and only SBP hypotension in post-exercise were observed, this suggested that post-exercise hypotension does not impact upon the stress-related BP response itself. Similarly, previous studies [18,34] observed reduced BP reactivity post-exercise, regardless of BP hypotension post-exercise.

A recent meta-analysis showed that acute PE lowers SBP and DBP reactivity in response to stressor tasks, including the SCWT, representing average reductions of ~4 and 3 mmHg, respectively, for SBP and DBP [4]. Overall, acute exercise particularly of the moderate-intensity aerobic kind reduces stress-induced BP reactivity in the general population [7]. The interval between the mental stress test and the end of the physical exercise session (ranging from 20 to 60 min) was the most controversial factor among the studies reviewed [18,33,34]. Moreover, these findings cannot be generalized to represent a full day of stress reduction during teaching activities. To address this, future research employing this experimental design is warranted. One study [6], investigating the effects of exercise performed prior to occupational teaching activities, verified that exercise induced a BP reduction in university professors, with the main effects being observed during subsequent teaching and sleeping hours, showing that a single exercise session helped control BP during stressful situations or the occupational activities of daily living.

The mechanism by which stress increases BP can be associated with aldosterone reactivity, which is higher in healthy hypertensive than normotensive [35] people. In addition, the mechanisms by which PE moderates mental stress-induced responses are still uncertain. HR reactivity to stress in our study was not reduced by acute PE, like in previous studies, [18,34]. These studies suggest that lower reactivity BP post-exercise was due to vascular resistance reduction [5]. Brownley [36] showed that reduced BP responses to a stress task (mental arithmetic task) after acute exercise were strongly linked to decreased sympathetic drive, evidenced by reduced norepinephrine responses. In addition, in cardiac and vascular β-adrenergic receptors (β1- and β2-receptor), responsivity increased indicating that the BP response was primarily blunted by enhancing β2-mediated vasodilatation. Furthermore, reduced vascular resistance during exposure to a stressor was observed on the exercise day compared to the control day [37]. Furthermore, hemodynamic stimulation and increased shear stress on vasculature due to exercise [38] can upregulate vasodilatory mechanisms within the exercising muscles and in the systemic circulation [5] and as a result, the BP reduction.

This study shows ecological validity, because the PE session, SCWT, and cardiovascular variables performed are simple and low-cost and can be easily carried out in a school environment. However, some limitations must be considered: (1) the small number of teachers evaluated due to the strict exclusion criteria necessary to analyze adequately the BP response to mental stress; (2) the level of physical activity was estimated through IPAQ and not objectivity measured, i.e., with an accelerometer; (3) the teacher’s psychological health was not considered; (6) the teacher’s sedentary behavior was not measured; (5) although we did not perform a control session, all cardiovascular reactivity variables to SCWT were reproducible (according to our pilot study). So, it is unlikely that the decreased BP reactivity response to mental stress is due to experience of the SCWT test at baseline time. The BP and HR during SCWT can be used to assess cardiovascular reactivity and to analyze the effect of different interventions on these variables.

Teachers need to have good physical and mental health to meet the care requirements of their students. The teacher’s health must be a concern of the institution and can reflect on service performance. Future intervention strategies with school teachers should consider these issues. Among the practical applications, we encourage the performance of physical exercise before the teachers’ daily work routine, which might be an interesting strategy for cardiovascular protection. Also, schools and educational institutions should consider incorporating regular physical activity programs for teachers to manage occupational stress and reduce the risk of hypertension. Finally, we suggest future research involving different types, intensities, and durations of physical exercise, a longer time (>1 h) between the mental stress test and the end of the physical exercise session, as well as the influence of physical activity levels on the stress response in this population. Also, future research should investigate the long-term effects of regular physical exercise on BP reactivity and the overall cardiovascular health of teachers.

## 5. Conclusions

In summary, SBP and HR reactivity to mental stress correlated negatively with obesity markers. Moderate-intensity aerobic exercise moderates BP reactivity to mental stress in teachers. Our findings are important for defining adequate clinical approaches for the prevention and non-pharmacological treatment of obesity and hypertension, aiding teachers to maintain adequate physical and mental health to perform their occupational tasks.

## Figures and Tables

**Table 1:** Cardiovascular reactivity to Stroop color word test in test and retest.

Variable (n = 12)	Time	Test	Retest	*p*	ICC	SEM	CV (%)
Systolic BP (mmHg)	0 min	105 ± 12	105 ± 11	0.84	0.94	2.88	10.68
	2 min	111 ± 14	110 ± 12	0.49	0.87	4.84	11.30
	4 min	112 ± 14	112 ± 12	0.94	0.81	5.96	11.54
Diastolic BP (mmHg)	0 min	63 ± 10	65 ± 10	0.07	0.92	2.57	13.67
	2 min	71 ± 9	70 ± 11	0.43	0.91	2.98	11.96
	4 min	71 ± 8	70 ± 10	0.79	0.90	2.99	10.07
Heart rate (bpm)	0 min	65 ± 16	65 ± 12	0.21	0.82	6.25	21.33
	2 min	72 ±13	70 ± 13	0.10	0.94	3.00	17.19
	4 min	73 ± 11	71 ± 12	0.21	0.91	3.40	14.87

BP: blood pressure; CV: coefficient of variation; ICC: intraclass correlation coefficient; SEM: standard error of measurement.

**Table 2:** Obesity and hemodynamic markers, classification (%) of nutritional status, blood pressure and physical activity level of teachers.

n = 31	
Sex (%women)	67.74
Age (years)	39.41 ± 7.07
Body mass (kg)	73.85 ± 12.23
Body mass index (kg·m^−2^)	26.03 (21.30–39.90)
Abdominal circumference (cm)	89.97 ± 11.67
Body fat (%)	36.47 ± 9.40
Systolic BP (mmHg)	114 ± 10
Diastolic BP (mmHg)	76 ± 9
SD1 (ms)	17.25 (8.55–45.91)
SD2 (ms)	43.45 ± 12.98
RMSSD (ms)	24.37 (12.07–64.82)
SDNN (ms)	34.00 ± 10.32
pNN50 (%)	3.70 (0–51.48)
HF (n.u.)	31.54 ± 18.56
LF/HF	2.72 (0.20–15.02)
Classification	(%)
Normal weight	35.48
Overweight	48.39
Obese	16.13
High abdominal circunferemce (women > 80 and men > 94 cm)	58.06
High BP (systolic BP ≥ 130 or diastolic BP ≥ 85 mmHg)	19.35
Physically active	67.74
Insufficiently active	32.26

BP: blood pressure; HF: high-frequency; LF/HF: low- and high-frequency ratio; n.u.: normalized units; pNN50: the relative number of successive RRi that differed in duration more than 50 ms; RMSSD: the root mean square of successive RR differences, SDNN: the standard deviation of normal-to-normal RRi; SD1: standard deviation of the instantaneous RRi; SD2: standard deviation of the long-term RRi. Parametric data are presented as mean ± standard deviation. Nonparametric data are presented as median (minimum–maximum values).

**Table 3:** Physical exercise indicators.

n = 31	
Heart rate (bpm)	132 ± 8
Distance performed (km)	3.02 ± 0.37
Velocity (km·h^−1^)	5.99 ± 0.73
Rate of perceived effort	10 (7–13)

Parametric data are presented as mean ± standard deviation. Nonparametric data are presented as median (minimum–maximum values).

**Table 4:** Cardiovascular responses to Stroop color word test at baseline and after acute exercise.

						Two-ANOVA		
	Time (min)	0	2	4		Time	Condition	Time × Condition
Systolic BP (mmHg)	Baseline	114 ± 10 ^a^	129 ± 15 ^b^	127 ± 17 ^b^	*p*	<0.01	<0.01	<0.01
					ES	0.53	0.48	0.13
	Post Exercise	111 ± 10 ^a#^	121 ± 15 ^b#^	119 ± 17 ^b#^	Pr	1.00	1.00	0.76
Diastolic BP (mmHg)	Basal	76 ± 9 ^a^	86 ± 10 ^b^	85 ± 10 ^b^	*p*	<0.01	0.10	0.01
					ES	0.45	0.09	0.19
	Post Exercise	78 ± 10 ^a^	84 ± 11 ^b#^	81 ± 12 ^b#^	Pr	1.00	0.37	0.92
Heart rate (bpm)	Baseline	77 ± 9 ^a^	85 ± 10 ^b^	85 ± 8 ^b^	*p*	<0.01	<0.01	0.55
					ES	0.58	0.54	0.02
	Post Exercise ^#^	85 ± 11 ^a^	91 ± 11 ^b^	92 ± 11 ^b^	Pr	1.00	1.00	0.15

BP: blood pressure; ES: effect size; Pr: statistical power to posteriori. Different letters indicate significant differences between times (0, 2 and 4 min) during the Stroop color word test. ^#^ Significant difference between post-exercise and baseline conditions.

## Data Availability

The data that support the findings of this study are available from the corresponding author upon reasonable request.

## Abbreviations

The following abbreviations are used in this manuscript:

BMI	body mass index
BP	blood pressure
CV	coefficient of variation
DBP	diastolic blood pressure
HR	heart rate
HRV	heart rate variability
ICC	intraclass correlation coefficient
IPAQ	International Physical Activity Questionnaire
PE	physical exercise
SBP	systolic blood pressure
SCWT	Stroop color word test
SEM	standard error of measurement
